# Description of the nationally implemented National Health Service digital diabetes prevention programme and rationale for its development: mixed methods study

**DOI:** 10.1186/s12913-023-09210-3

**Published:** 2023-04-18

**Authors:** Lisa M Miles, Rhiannon E Hawkes, David P French

**Affiliations:** Manchester Centre for Health Psychology, University of Manchester, Oxford Road, Manchester, M13 9PL United Kingdom

**Keywords:** Diabetes prevention, Digital interventions, Fidelity, Support, TIDieR, Behaviour change

## Abstract

**Background:**

The National Health Service (NHS) Digital Diabetes Prevention Programme (DDPP) is a behaviour change programme for adults in England who are at high risk of developing type 2 diabetes. Four independent providers deliver the NHS-DDPP following a competitive tendering process. Although providers work to a single service specification, there is potential for some variation in the service across providers. This study (1) assesses fidelity of the structural features of the design of the NHS-DDPP compared to the service specification, (2) describes the structural features of delivery of the NHS-DDPP as implemented (3) reports developers’ views on how the structural components of the NHS-DDPP were developed and why changes were made following implementation.

**Methods:**

Using mixed methods, we conducted a document review of providers’ NHS-DDPP design and delivery documentation, and extracted information using the Template for Intervention Description and Replication checklist, which was adapted to capture features of digital delivery. Documentation was supplemented by content analysis of interviews with 12 health coaches involved in delivering the NHS-DDPP. Semi-structured interviews were also conducted with 6 programme developers employed by the digital providers.

**Results:**

Provider plans for the NHS-DDPP show relatively high fidelity to the NHS service specification. Despite this, there was wide variation in structural features of delivery of the NHS-DDPP across providers, particularly for delivery of ‘support’ (e.g. use, dose and scheduling of health coaching and/or group support). Interviews with developers of the programmes showed that much of this variation is likely to be attributable to the origin of each provider’s programme, which was usually a pre-existing programme that was adapted to conform to the NHS-DDPP service specification. The NHS-DDPP is continually improved and developed based on user experience feedback and research conducted by the providers.

**Conclusion:**

Indirect evidence suggests that variation in delivery of support could affect effectiveness of the NHS-DDPP. A priority for future research is ascertaining whether the variation in delivery of the NHS-DDPP across providers is related to any differences in health outcomes. It is recommended that future rounds of commissioning the NHS-DDPP pre-specify the type of support participants should receive, including expected dose and scheduling.

**Supplementary Information:**

The online version contains supplementary material available at 10.1186/s12913-023-09210-3.

## Background

Type 2 diabetes mellitus (T2DM) is a major public health concern that is largely preventable by weight loss and improved diet and physical activity. Following the success of diabetes prevention trials [1–3], several diabetes prevention programmes have been implemented around the world [4, 5].

In 2016, the National Health Service (NHS) in England implemented the Healthier You: NHS Diabetes Prevention Programme (NHS-DPP) for adults at risk of developing T2DM. The NHS-DPP is a behaviour change programme delivered in groups which aimed to support participants improve dietary and physical activity behaviours, and prevent progression to T2DM. It has been rolled out in waves, gradually reaching universal national coverage [6]. Delivery of the NHS-DPP is procured through a national competitive process, organised by NHS England (NHSE); this was originally conducted through a Framework Agreement in 2016 (Framework 1), in which four providers were selected to deliver a face-to-face version of the programme [7].

Early favourable health outcomes from the NHS-DPP have been reported [6, 8], but it has been suggested that measures are needed to improve uptake and retention on the programme for younger people, those in employment, minority ethnic and deprived groups and those reporting a disability [9]. A digital version of the NHS-DPP was piloted in 2017/2018; participation in the programme was associated with clinically significant reductions in weight (-3.1 kg) and HbA1c (-1.6mmol/mol) at 12 months [10]. These changes have been shown to be comparable to those of the face-to-face programme [11].

The NHS-DPP was then re-procured in 2019, when a number of enhancements were made to the service specification, including the introduction of a digital DPP service as an adjunct to the face-to-face service (Framework 2). At this point, five providers were selected to deliver the face-to-face group service by NHSE to provide NHS DPP across England: four of the previous providers plus one new provider [7]. Four of these five providers sub-contracted digital providers to deliver the digital service (NHS-DDPP). This resulted in important partnerships between providers of the face-to-face and digital programmes. As part of the competitive process to secure contracts to deliver the programme, face-to-face and digital providers worked together to submit a Framework 2 response describing their proposed service delivery and planned content. During delivery of the service under Framework 2, one digital provider contract ended. The remaining four digital providers are commercial entities.

NHSE produced a Framework 2 service specification [12] detailing the key features that should be present in the NHS-DPP [12] based on the currently available evidence [13, 14]. The present research examines the fidelity of each digital providers’ programme to this specification. Intervention fidelity refers to whether an intervention was designed, delivered and received as planned [15]. An assessment of fidelity is important to fully understand the reasons why interventions are effective or not. Without it, reports of an effective intervention could be a function of either an effective intervention or the influence of other unknown factors added to or omitted from the intervention [16]. Our research team has reported extensively on the fidelity of the behaviour change content of face-to-face and digital versions of the NHS-DPP [17–20].

In the present study we assess the fidelity of the structural features (such as materials and mode of delivery) of the design of the NHS-DDPP compared to the NHS service specification, and describe the delivery of the NHS-DDPP using the Template for Intervention Description and Replications (TIDieR) checklist [21]. The TIDieR checklist has been developed as part of a movement towards standardised reporting of non-pharmacological interventions. Accurate description of interventions is important to facilitate replicability and implementation beyond randomised controlled trials (RCTs).

In addition to an assessment of fidelity to the service specification, it is also important to fully document the reasons for changes in key structural features of delivery of the NHS-DDPP as it is implemented. Although the programme is commissioned centrally by NHSE and each provider is working to a single service specification, because the programme is delivered by multiple providers, there is potential for some variation in the service across providers. It is important to understand what (if any) variation occurs as the programme is implemented. Further, it is informative to explore the journey by which the provider programmes were developed, to explain how and why the programmes were structured as they are. Such findings can provide important context for further evaluative work on the NHS-DDPP and further inform implementation of the NHS-DPP and other similar programmes.

A mixed methods approach has been chosen to document the structural features of the NHS-DDPP (quantitative) and the evolution of the provider programmes throughout the journey of development and implementation (qualitative). Specifically, the objectives of the present study are firstly to: (1) compare the structural features of the NHS-DDPP design to the NHS service specification for the programme (fidelity); (2) describe the structural features of the delivery of the NHS-DDPP using TIDieR (highlighting variation across providers and any modifications introduced following implementation). Secondly, a series of interviews with NHS-DDPP programme developers has allowed a qualitative assessment of (3) how the structural components of the NHS-DDPP were developed and why changes were made to the programme following implementation.

## Methods

### Design

This study used a mixed-methods design. Components of the NHS-DDPP were content-analysed for structural features of design (objective 1) and delivery (objective 2). Semi-structured interviews investigated how the NHS-DDPP was developed and any rationale for any changes made to the NHS-DDPP once implemented (objective 3).

### Document review

#### Programme specification documents

The programme specification documents for the NHS-DDPP indicate the key content that should be included in the programme, and comprise of the NHS service specification [12] and NICE Guideline PH38 on Type 2 Diabetes: Prevention in People at High Risk [14]. The former was specific to commissioning of the NHS-DPP (including face-to-face and digital offerings in Framework 2) and was based on an evidence review of lifestyle interventions for the prevention of T2DM [13] and drew on recommendations from NICE PH38 guideline [14]. The NICE PH38 guideline provided additional information regarding behaviour change content to be included in diabetes prevention programmes and was referred to in the NHS Service Specification [12]. Key structural features from these specification documents have been identified in a previous study [22] and have been used again here as a basis for assessing fidelity in the current study.

#### Design materials

The design documentation supplied by each digital provider to the research team has been described previously [17]. In brief, this comprises Framework 2 response bids each provider submitted to NHSE (by 15th October 2018), during procurement and supplementary information (further documentation and/or email correspondence) obtained from providers between June 2020 and April 2021.

##### Analysis

The design of each provider version of the NHS-DDPP was described using the TIDieR checklist [21]. TIDieR items (e.g. materials, procedures, modes of delivery) were extracted by REH from the Framework 2 responses; TIDieR items were later extracted by LMM from supplementary design information from providers. The final TIDieR description was checked with the service provider for accuracy. Results were tabulated and compared with requirements in the NHS specification as an assessment of fidelity.

#### Delivery materials

The delivery documentation provided by each digital provider to the research team has been described previously [20] and is detailed in Additional file 1. This comprised:

- Guest access to smartphone and web applications for three out of the four providers. The other provider supplied an app user guide.
- All educational materials, including learning platforms, online articles, PDF articles, videos, online workbooks and additional workbooks posted to service users.
- Standard text/script sent to service users via email and text messages.

##### Interviews with health coaches

Further, a series of semi-structured interviews were conducted with health coaches who are actively involved in delivering the NHS-DDPP. A total of two, four, two and four interviews (n = 12) were conducted with health coaches from providers 1, 2, 3 and 4 respectively between July and November 2021. Recruitment and informed consent procedures for health coaches taking part in the interviews are described elsewhere [20]. In brief, interviews were conducted via the video conferencing platform Zoom and covered the following topics of relevance to the present study:

- Participants’ professional background and any training received.
- Participants’ role in supporting service users throughout the programme (e.g. at first contact, continued engagement, coaching via telephone/video calls and/or online chat, moderating support forums).
- Participant’s role in tailoring or personalising coaching to individuals.
- Content of the digital intervention, including the format of intervention features included in the programme and any modifications that have been made.

The full topic guide for the interviews is available in Additional file 2.

##### Analysis

Although the TIDieR checklist [21] has been found to be a useful tool for applied health research studies [23], it was originally developed before the rapid growth in availability and application of *digital* health interventions. We therefore considered recent literature relevant to describing digital health interventions [24–26] and adapted the items in the TIDieR checklist to better reflect description of a nationally implemented digital health intervention (NHS-DDPP). Specifically, items 6 (‘how’), 8 (‘when and how much’) and 9 (‘tailoring’) have had sub- items added to carefully describe format of delivery, details of scheduling of different procedures (information sessions and health coaching) and nature of tailoring. These changes are largely built on a framework for form of delivery (which includes all features through which behaviour change intervention content is conveyed including: the provider, format, materials, setting, intensity, tailoring, and style) [26] and informed by important aspects of delivery highlighted in an ontology for specifying the mode of delivery of interventions [24] (namely, adding 'interactivity’). In addition, we acted on a recommendation [23] to include a column in the TIDieR checklist for ‘modifications’, so that changes to the programme since implementation could be captured (see Additional file 3 for adapted TIDieR checklist). Further, we decided to not report rationale, theory and goals of the NHS-DDPP from item 2 (‘why’) of the TIDieR checklist in the current study, as this has already been reported in previous work by the research team regarding theoretical underpinnings of the NHS-DDPP [17]. In addition, item 7 (‘where’) of the checklist was removed as this was not relevant in a digital context.

The adapted TIDieR checklist was used by LMM and REH to extract relevant information on delivery of each provider version of the NHS-DDPP. This included content analysis of all delivery documentation listed in Additional file 2 and transcripts of 12 interviews with health coaches, and extracting relevant excerpts into a series of adapted TIDieR checklists. Triangulation was then conducted across these multiple sources of information and results were tabulated. Tables were then reviewed to assess variation across providers and to highlight modifications that had been made to the provider programmes during implementation. The final TIDieR description was checked with the service provider for accuracy.

### Qualitative interviews of programme developers

#### Participants

Interviews were conducted (by LMM and REH) between September and December 2020 with programme developers employed by each of the four digital providers. Programme developers were involved in the design and development of the NHS-DDDP and/or were a key contact at the digital provider best placed to describe how the content was developed for the programme. Interviews with two professionals took place with digital providers 2 and 3, and interviews with one professional took place with digital providers 1 and 4 (n = 6 interviews in total). Further details on procedures for informed consent and recruitment are reported elsewhere [17].

#### Topic guide

The interviews were semi-structured and covered a range of topics, including theoretical underpinnings of their programmes, planned behaviour change content and strategies to support engagement; the results of which are published separately [17]. The full topic guide is available in Additional file 4. Interview topics of relevance to the present study included:

- The process of developing and/or adapting the NHS-DDPP programme content, including the extent to which the programmes were adapted from any pre-existing digital programmes.
- The relationship between the NHS-DDPP with that of the partner face-to-face offering of the programme.
- Content of the digital intervention and format of the different intervention features included in the programme.

Interviews were conducted by the video call platform Zoom. Recordings were transcribed verbatim.

#### Analysis

Transcripts were analysed thematically using Nvivo software (version 12). Full transcripts were reviewed for familiarity and then sections of the interviews relevant to research objective 3 were coded inductively (by LMM). Once all coding was completed, themes were initially generated by LMM. Theme descriptions were then discussed and refined further by all authors.

## Results

### Fidelity of design of digital provider programmes compared to service specification

Overall fidelity of the structural features of each digital provider’s programme in comparison to those outlined in the NHS service specification (based on providers’ Framework response documents and other supplementary design documentation provided to the research team) was relatively high (Table 1). Duration and frequency of the programme was in line with the requirements of the specification. The ‘mode of delivery’ and ‘materials’ requirements in the service specification were focussed more on the face-to-face format of delivery, and so some variation in how ‘sessions’, materials or content was planned to be delivered digitally is to be expected. Each provider planned to offer ‘support’ to participants and allowed ‘tailoring’, though the mode of delivery of such support and tailoring varied across providers. Routes for measuring participants’ bodyweight and HbA1c were planned for all provider programmes, with the exception of provider 4 that did not refer to HbA1c measurements in its programme plans.

**Table 1:** Service parameters outlined in the programme specification in comparison to each digital provider’s design documentation

	NICE PH38	NHS Service Specification	Provider 1	Provider 2	Provider 3	Provider 4
**Initial assessment**	-	Adequate information on benefits and risks of service to allow informed decision to participate, confirm eligibility, baseline data, brief intervention for smoking	Telephone call, confirm baseline data, questions answered, brief interventions (including for smoking cessation where appropriate), technology explained, commitment to behaviour change, Healthbox sent in post	After mini assessment to take weight and HbA1c 60-minute onboarding F2F video call to establish relationship, commitment to behaviour change, set initial goals	Pre-assessment surveys^3^, email, SMS or telephone call (45-minutes^3^) to confirm eligibility, brief interventions including for smoking, deliver motivational interviewing, discuss weight measurement options	Targets set for weight loss, 30-minute onboarding call with coach, access to educational content, motivational interviewing. Smoking cessation advice available.
**Duration**	9–18 months with follow-up sessions for two years	Minimum of 9 months	9-month programme divided into 2 phases	9-month programme	9-month programme, divided into 3 phases^3^	13 curriculum topics over 9-month programme
**Frequency**	Tapered and delivered in a logical progression	Allow sufficient time between sessions to make gradual behaviour changes, engagement activity each month	Core phase (weeks 1–12): new content unlocked daily. On demand coaching with average 2-hours dedicated coach time per week via messages. Sustain phase (weeks 13–40): new content unlocked weekly. On demand coaching with average 30–60 min of coaching time per week. Frequency and intensity of coach support reduces over time.	2 personal coaching sessions per month over 9-months (but can be front-loaded to support initial motivation), 16 2-minute bite-sized videos and written modules delivered to enhance coaching support	Modular curriculum unlocked each 30 day period, frequency of HC prompts and guided feedback gradually reduced from weekly to monthly ^3^Start/Core phase: intensive weekly app coaching for 6 weeks (3 times per week), or fortnightly phone calls. ^3^Sustain phase: monthly calls from HC for 6 months	Prompted to engage with tools and curriculum > fortnightly ^2^Behaviour change techniques delivered weekly
**Mode of delivery**	Individual or group sessions	13 sessions must be provided in a format appropriate to a range of diverse groups	Access to app/ web-application, notifications via email, text and ‘push’ messages	Content of sessions include messages, videos, pdf’s, links, education offered in both written and video content	Delivered by smartphone app, online, or as a phone-based service. Articles, videos, podcasts material online, or available offline in pdf/printed.	Access to app/ web-application, audio, video and interactive content, structured education via email, programme prompts through emails and texts, materials can be in print or electronic
**Materials**	The programme should offer practical learning opportunities, particularly for those who have difficulties with communication and literacy	The programme material designed to allow service users with different levels of knowledge and different approaches to learning to progress at different paces, promoting self-directed learning	Wireless scales, handbook, activity tracker, recipe book, articles, action plans, tracking tools on app, articles, online exercise videos	Tracking and goal setting tools, structured education videos/ pdf’s	Online learning portal, optional Guidebook/DVD if no online access. Includes meal plans, recipe book, articles, videos, podcasts, quizzes, tracking tools	Weekly videos and email content, workbook, > 1,000 recipes, barcode scanner, tracking tools, content from Headspace and Aaptive^a^, nutritional data for branded foods, behaviour-based rewards, articles detailing ‘success stories’
**Support offered**	Support from sensitive, well-trained and dedicated people; encourage support from family members	Consider social and psychological support needed to support people to implement behaviour changes, provide individual 1:1 support	Personalised health coaching through 1:1 messaging, participant peer support groups of approximately 10 people, average total coach time via messenger is 36 + hours per service user	60-minute onboarding video call, 18 personal coaching sessions, algorithmic messaging support, access to online community groups (grouping based on interests, etc.), same HC throughout programme	Initial call with HC to set action plan for diet, physical activity and weight loss, meal plan designed, at least 10 1:1 support sessions from HC via telephone and messaging/ video calls, peer groups support of 10 service users, option for carer/ relative to join 1:1 calls, same HC throughout programme	Telephone support at initial assessment, access to social community groups (connect), 24/7 1:1 chat
**Tailoring**	Sensitive and flexible to the needs, abilities, and cultural or religious norms of local people	Tailored to personal circumstances and culture of service users, sensitive to different culinary traditions, maximise flexibility of offering	Tailored coach advice for setting diet and activity goals, nutritional advice specifically for different cultural backgrounds, and individualised support^1^.	Individualised adaptable goal setting, mode of educational resources personalised to user (e.g. videos, pdf’s)	Physical activity advice graded and structured within personalised timeframe, learning materials personalised to level of knowledge and approach to learning, multi-lingual dieticians/HCs for over 15 languages, personalised meal plans for cultural and culinary traditions, varying appointment times offered (8am-8pm Monday-Saturday) ^3^Health Action Process Approach tool completed after the first 2 weeks of coaching to help determine whether coaching or self-led approach.	Range of different channels to support different learning styles/ abilities/ cultural needs, support materials in > 10 languages, private online groups for like-minded people, recipes to suit all culinary traditions, personalised goals, booked calls with translators
**Measurements (weight, HbA1c)**	-	Regular weigh-ins (self-monitoring); baseline, 3-month, 6-month and 9-month weigh-ins taken via calibrated mechanisms. Blood test at first and final session	Provided with wireless scales to record weights at IA, and months 3, 6 and 9, at weeks 37–40 blood test is taken by F2F provider	Service users initially invited to blood and weight testing mini-assessment at a F2F session or pharmacy	Weekly self-monitoring of weight encouraged. Weight measurements and HbA1c taken at F2F measurement clinics, or local weight machines, validated weight at days 30, 90, 180 and 270	Weight measurements at F2F groups or pharmacy

^1^Individualised support identified in additional design documentation, dated 09/2018, and supplied to research team on 09/02/2021

^2^Frequency of behaviour change techniques identified in additional design documentation, received 08/04/2021

^3^Further information identified in additional design documentation (dated 10/06/2020). This included an indication that initial phone call was 30 min duration rather than 45 min

HC = Health coach

F2F = Face-to-face

#### Structural features of the delivery of the NHS-DDPP using the adapted TIDieR checklist

According to providers’ delivery materials supplied to the research team between July 2020 and August 2022, and content analysis of interviews with health coaches conducted in 2021, programme content across providers is similar in terms of the use of three main categories of content: use of a smartphone app, educational material and support (Table 2). In particular, programme content delivered by a smartphone app was relatively consistent across providers; for example each app had functions to facilitate tracking of behaviours and/or outcomes.

**Table 2:** The structural features of four provider programmes delivering the NHS-DDPP, including modifications identified

		Provider 1	Modifications	Provider 2	Modifications	Provider 3	Modifications	Provider 4	Modifications
Materials	Virtual materials	App including tracking, chat, toolbox and activity functions; 12 weeks of Core Learn educational materials; Option to sign up to 7 Sustain course modules		App including tracking, personal profile, ‘agreement’, messages and group functions; 41 pdfs and 13 videos of educational material	HC dashboard of available pdfs/videos is growing	App including goal setting ‘to dos’, tracking weight & behaviours and graphs of progress; chat with coach/peers; access to online learning platform; and profile functions.		App including tracking, barcode scanner, food information and recipes; Access to educational materials (weekly techniques) via app; pdf of editable DPP workbook; pdfs on success planning and maintenance/signposting	
	Physical materials	Handbook (Introduction, Nutrition advice, Meal planning, Q&A); Tracker; Scales				Printed guidebook available if no online access			
Procedures	Educational material	Core Learn articles for weeks 1–12; links to exercise videos, recipes; Option to sign up to 7 Sustain course modules	Content of articles continually reviewed	Series of pdfs and videos sent to participant by HC		Structured Learn platform comprising 42 lessons, gradually unlocked over 9 mo.	Content of modules is continually reviewed and updated	1 pdf editable workbook sent to participants at start of programme; 1 pdf workbook to ‘plan success’; 1 pdf sent to participants at end of programme with focus on signposting; Weekly articles available via the app	
	App	App includes procedures to plan meals, set habits, participate in steps leaderboard, track weight, steps and sleep (device can be paired with app), enter reflections (food diary and journal)		App includes procedures to set and track progress against goals, track multiple behaviours and outcomes, and view agreement between HC and participant		App includes procedures to set and track goals ‘to do’s, track diet, physical activity and steps (fitness tracker devices can be synced with app), weight and other outcomes		App includes procedures to track activity, sleep, water and food; assess nutrition information of foods, plan meals and access workout videos and audio coaching for mindset. Series of emails sent to participants to encourage use of app functions	Changes to points system for monitoring progress and personalised food plans identified Nov 2021. Introduction of workout videos and mindset coaching (based on new research and user feedback in 2020)
	Support	First 12 weeks: 1–1 messaging with HC and closed group chat. After week 12 Community Groups discussion forums available (choice of 17 topics) (post, like and comment); Sustain chat group -participants in each 4 week course added to the relevant module ‘chat’ group. Closed group chat (peer-peer) avail all 9 Mo, but only moderated by HC up til week 24. HC available to respond to private 1–1 messages for 24 weeks		Initial consultation by live video call, followed by series of asynchronous video messages (with text), and pdfs/videos sent from HC to participant. HC use signposting to other resources and recipes. Group support (optional): group forum for peer support (post, like, comment, not actively moderated by HC)		Initial consultation telephone call, followed by app or phone coaching (scheduled), then monthly telephone calls. Discharge telephone call at end of programme	Choice of 3 pathways introduced in July 2021: Group coaching pathway (20 members moderated by HC); App coaching pathway; Phone coaching pathway	1–1 messaging available with access to online HCs 24/7. Group support forum (post, like, comment, HC present)	
Format of delivery (per procedure)		**Core Learn articles**		**Pdfs**		**Learn platform**		**Workbook and pdfs**	
	Mode of delivery	Remote		Remote		Remote		Remote	
	Delivery method	Individual		Individual		Individual		Individual	
	Delivery channel	App/website (*Passive)*		App, chat *(Passive, potential for prompted interaction).*		App/website *(Passive)*		Website/email *(Passive)*	
	Delivery route	Text, audio, video		Text, picture		Audio, text, picture, video		Text, picture	
		**Sustain modules**		**Videos**				**Weekly articles (techniques) via app**	
	Mode of delivery	Remote		Remote				Remote	
	Delivery method	Individual		Individual				Individual	
	Delivery channel	App, email *(Passive)*		App, chat *(Passive, potential for prompted interaction).*				App, email *(Passive)*	
	Delivery route	Text		Audio, video				Text, picture, video	
		**App**		**App**		**App**		**App**	
	Mode of delivery	Remote		Remote		Remote		Remote	
	Delivery method	Individual		Individual		Individual		Individual	
	Delivery channel	App (*Interactive)*		App (*Interactive)*		App (*Interactive)*		App (*Interactive)*	
	Delivery route	Experiential		Experiential		Experiential		Experiential	
		**Support (group chat and community groups)**		**Support (group forum)**			**Support (group chat) introduced in July 2021**	**Support (group forum)**	
	Mode of delivery	Remote		Remote			Remote	Remote	
	Delivery method	Group (group chat up to 20 people)		Group			Group	Group	
	Delivery channel	Chat (*Interactive)*		App, chat *(interactive)*			App, chat *(interactive)*^*1*^	App, chat *(interactive)*	
	Delivery route	Text, picture		Text, picture			Text, picture^*1*^	Text, picture, video	
		**Support (health coaching)**		**Support (health coaching)**		**Support (health coaching)**	**Revised July 2021**	**Support (health coaching)**	
	Mode of delivery	Remote		Remote, remote and face-face		Remote (remote face-face option available)	Remote (remote face-face option available)	Remote	
	Delivery method	Group, individual		Individual		Individual	Individual/group	Individual	
	Delivery channel	Chat *(Interactive)*		Video call, chat, video messaging *(interactive)*		Telephone, chat *(interactive)*	Telephone, chat *(interactive)*	Chat, (occasionally telephone) *(interactive)*	
	Delivery route	Text, pictures		Text, audio, video, picture		Text, audio	Text, audio	Text, audio	
		**Physical materials**							
	Mode of delivery	remote							
	Delivery method	Individual							
	Delivery channel	Physical booklet and tracker (passive)							
	Delivery route	Text, images (handbook); unclear (tracker)							
Deliverers^2^		Undergraduate/postgraduate degree in Psychology/Nutrition; further training in coaching, safeguarding; CBT		Undergraduate/postgraduate degrees in Sports Science and Clinical Exercise Physiology; further training including motivation, behaviour change; Hospitality/nutrition adviser background; Clinical medicine background; further training including motivational interviewing, behaviour change		Undergraduate/postgraduate degree in nutrition; further training including motivational interviewing, behaviour change, patient trauma, medical conditions, diabetes, information governance		Previous members of programme, further training in behaviour change, diabetes	
Dose and scheduling	Sessions/educational material	Core Learn material unlocked in weeks 1–12. After 12 weeks: Sustain modules take place over 4 weeks, with 3 articles per week sent Mon/Wed/Fri		Pdfs and videos sent to participants by HC according to need over 9 mo (approx 2 resources sent per interaction)		42 weekly lessons, gradually unlocked over 9 mo.		Series of emails sent weeks 1–4 (4 per week) to encourage use of all programme procedures e.g. tracking, meal planning	
	Health coaching	Group chat: HC makes contact proactively daily for first 12 weeks, proactively weekly for next 12 weeks, then leaves group. Community groups: HC makes contact proactively weekly. Private chat: Proactive check-in by HC 3 x in first 12 weeks, then reactive to Qs up to week 24.		Initial consultation with HC by live video call (45–50 min) at start of programme; subsequent asynchronous HC video messages (3 min) / interventions (lasting 9-10 min) weekly for first 3 months, bi-weekly for next 3 months and monthly for final 3 months.		Initial consultation with HC by telephone (30 min) in week 1; followed by six weeks of app coaching (HC proactive 3 times/week, 5 min each time) or three bi-weekly phone calls (20-30 min). Remainder of programme to 9 months phone coaching from HC at pre-agreed time points (monthly telephone calls of 20-30 min each)	Group coaching pathway: 45 min initial consultation with HC, followed by monthly 15 min group app interaction with HC 1–1 App coaching pathway: 45 min initial consultation with HC, followed by monthly 15 min 1–1 app interaction with HC 1–1 Phone coaching pathway: 45 min initial consultation with HC, followed by monthly telephone calls with HC (30 min each) Telephone coaching sessions reduced from 30 to 20 min in July 2021	HC support is provided reactively to participant questions (not scheduled)	
	Support (peer-peer)	Group chat remains open for peer-peer chat for 9 mo		Group forum available for 9 mo				Group forum available for 9 mo	
Tailoring	Automated			Series of scheduled ‘Support’ emails sent to participants who do not engage with programme				Messages of encouragement sent to participants when they log their weight; automated tailoring of goal setting on app and feedback on tracked information	
	Tailored by whom?	HC can tailor app experience (e.g. Leaderboard on/off, feedback on food diary) and support according to lifestyle factors e.g. working patterns, caring responsibilities, disabilities.		HC tracked information, barriers to change, lifestyle		HC tailors advice and support according to individual assessment questionnaire which is completed prior to first appointment, tracked information and progress		HC can tailor advice when responding to questions e.g. setting of goals	

^1^Note information on format of delivery of group support, introduced by provider 3, was supplied by email correspondence rather than direct assessment of the app

^2^Note background and training experiences of deliverers based on interviews with a sample of health coaches, not on comprehensive training schedules/materials from providers

HC = Health coach; CBT = Cognitive behavioural therapy

Most importantly, there was wide variation in delivery of ‘support’ across providers, in terms of type (1–1 health coaching and/or group support) and for each type, further variation across delivery channel and method, and dose and scheduling. This highlights considerable variation in the intensity of coaching and support delivered to participants across providers. Provider 4’s health coaches are available to provide support reactively to participant questions and predominantly by online chat. In contrast, health coaches from providers 2 and 3 deliver initial consultations by telephone or video call for at least 30 min followed by a series of scheduled telephone calls (provider 3) or video messages (provider 2) over 9 months. Provider 1’s health coach support is predominantly offered in a closed group chat setting, and is proactively delivered by a health coach to the group (though private 1–1 chat also available) up to week 24 of the programme.

Group support was offered by three of the four providers during the period of data collection for this study. Again, variation was apparent in the delivery method across providers: two providers offered a group discussion forum (similar to Facebook) and one used a closed group chat (auto-enrolled into a group with functionality similar to Whatsapp) and there was variation in the length of time (if any) health coaches were available to moderate such groups.

There were further differences in the structural features of the programme across the four providers, in relation to deliverers of the programme (health coaches) and delivery channels/routes used by providers for educational material. The background of health coaches was consistent across providers 1–3, where they usually had at least degree level qualifications in a health-related subject, but health coaches from provider 4 did not have this.

To supply educational material, providers used a range of structured e-learning modules, emails, PDFs and workbooks, though on the whole the delivery channel was always passive (a one-way communication from provider to participant). Dose and scheduling of such information across the 9 months of the programme also varied widely.

A number of modifications to the provider programmes were identified (Table 2) including the introduction of a new choice of participant pathways for provider 3 in July 2021. This change included the introduction of an option for group support. Each of the four providers’ documentation referred to some degree of updated or review of materials or procedures, particularly for their educational materials, thereby showing that their portfolio of materials and procedures is not static.

### Qualitative interviews with programme developers

Two overarching themes were identified in the thematic analysis, which directly address research objective 3. Theme (1) *Adaptation of pre-existing programmes to meet NHS service specification requirements*, is broken down into three sub-themes that provide further explanation of the journey from pre-existing programmes to current versions of the NHS-DDPP. Theme (2) *Continuous development driven by user experiences* directly addresses why changes were made to NHS-DDPP following implementation.

#### Theme 1: adaptation of pre-existing programmes to meet NHS service specification requirements

Across interviews with all providers, it was clear that provider versions of the NHS-DDPP were not purpose-built from scratch. Each provider already had established experience in delivering digital healthcare interventions and had a pre-existing programme or programmes that could be adapted to meet the needs of the NHS service specification. For 3 out of 4 providers, such original programmes were usually a consumer-facing programme designed to help people lose weight and/or change health and wellbeing behaviours. These pre-existing programmes were then adapted in line with the specification, with key adaptations including adding material on prevention of T2DM and/or lengthening the programme. These were often described as ‘tweaks’ or additions rather than redesigns of a programme.

"What we kind of had to tweak really was that the NDPP was a bit more structured to what we offered because before we were very much a one-to-one personal coaching service, and every individual service would contain completely different information (Provider 2)".

"potentially we’re offering, obviously the usual service with additional curriculum. So when we get other people involved it’s far more from a point of view of how do we provide access codes to individuals rather than, let’s write a digital service for DPP… So when you’re asking for what our other – what other people inputted into it, for us it’s like we’ve got it, we need to adapt it for DPP but we’re not creating something new (Provider 4)".

#### Sub-theme 1.1 maintenance of ethos of original programmes

Furthermore, the ethos of the original programme was maintained in the NHS-DDPP programmes: *“yeah, we haven’t lost that initial, um, framework and philosophy, we’ve really just adapted it to fit the specification of what the commissioners have required (Provider 1)”*. The original ethos varied across providers (1, 2 and 4), including a focus on changing health and wellbeing behaviours, personal coaching targeting such behaviours and/or weight loss.

#### Sub-theme 1.2: relationship with face-face NHS-DPP programme

Providers of the NHS-DDPP are subcontracted by providers of the face-face DPP, with the exception of Provider 4 that delivers both digital and face-face versions of the DPP. Therefore the relationship with face-face providers was explored in the interviews, in terms of any influence on development of the NHS-DDPP. Responses were very mixed and varied across providers. Some digital providers described the development of their programme as completely independent of their face-face provider partner, for example:

“they didn’t have a huge amount of involvement in our product development I would say. We were given the specification of course, from the invitation to tender. But it was basically up to us to justify how we were going to meet that (Provider 1)”.

Provider 3 described some consultation with face-face partners about programme curriculum: *“I suppose when they displayed what topics they’d cover in each of their thirteen sessions, and we then equally displayed what we cover, actually we realised they were very similar in content wise and respects (Provider 3)”*.

Interviewees from providers 2 and 4 viewed the face-face and digital versions of the NHS-DPP as well integrated, particularly because participants in the face-face versions of the NHS-DPP also had access the DDPP apps.

#### Sub-theme 1.3: evolution from pilot version of DDPP

For those providers involved in the pilot study of the NHS-DDPP, experiences from the pilot study were an additional influence on the development of the version of NHS-DDPP implemented in Framework 2. For providers 1 and 2, this was viewed as an additional stage in the adaptation of a consumer-facing programme to the current version of the NHS-DDPP:

"So that’s – it’s developed – the intervention that we originally developed has evolved from a twelve-week programme to a six-month programme and to now a nine-month programme. And that’s the programme that we deliver to consumers and then adapting it to the digital pilot of the DPP and now, obviously, the current framework. So all of the different things that were on the specification that we needed to deliver on, and we had to adapt the intervention to make sure that we accommodated those and stood the best chance of winning a place in the framework (Provider 1)".

“it was mainly a case of kind of tweaking and localising the content of the delivery model to the UK of which the digital Diabetes Prevention Programme pilot was an initial framework for us to deliver that. Which was then modified based on feedback and tweaks to be the tender of framework for the national Diabetes Prevention Programme (Provider 2)”.

However, uniquely for provider 3, their programme was described as developed specifically for the pilot NHS-DDPP, based on experience of other digital health interventions including on digital T2DM management and weight management, and the experiences of the pilot prompted further development: *“So for us it was how did we support superior weight loss in the next phase of development with the national pilot. So we, the pathway was definitely adapted from the pilot because actually we could see the engagement worked (Provider 3).”* This provider further explained that their experiences of delivering other digital health interventions highlighted the need for intensive support in the early stages of the programme.

#### Theme 2: continuous development driven by user experiences

All providers described a process of continual improvement and development of their digital programmes. In this way, it is clear that any assessment of the NHS-DDPP captures a snapshot in time, as changes are introduced on an ongoing basis: *“we’re very proud of our app. It’s something that never stands still. It’s improved all the time (Provider 4)”; “I kind of have to emphasise that we do testing and iteration constantly, on a weekly basis (Provider 1).”*

These changes are largely driven by user experiences of the programme. Some providers described their own research methods to gain insights on user experiences: *“we’re running a trial at the moment to explore group chat function within the app……. So we’ll be doing user interviews and feedback, we’re collecting how many decline that, how many uptake that, and so we’re just trying to gather data now to see actually is that a good offering (Provider 3)”; “we’ve got our own kind [Provider] experience surveys so how patients find the app, what things they find useful, what they find not so useful. Or how they found the coaching, how was their relationship. So we’re constantly developing the app based on user feedback (Provider 2).”* Further, in some instances a periodic review of scientific recommendations and guidelines is conducted to ensure that the programme is routinely *“up-to-date and evidence-based (Provider 3)”.*

## Discussion

### Principal findings

Overall, the NHS-DDPP is a complex multi-faceted intervention, that requires substantial commitment from providers and participants. Our fidelity analysis of the NHS-DDPP shows provider plans for the programmes are generally in line with the NHS service specification. Important variation in programme features has been identified across the four digital providers, as well as modifications to the programmes since implementation. Important differences were identified in terms of support offered to participants (through health coaching and/or group support) and the delivery channels/methods and dose and scheduling by which such support was offered to participants. This results in substantial variation in intensity or support offered to participants across the four digital provider programmes.

Interviews with programme developers provided rationale and background to why such variation is apparent. For at least 3 out of 4 provider programmes, the origin was a pre-existing programme that was adapted to become in line with the NHS service specification for the DDPP, and much of the ethos of the original programme remained intact. Therefore, features of the programmes related to ‘support’ were more a reflection of the original programmes than what was required in the NHS service specification. As subcontractors of face-to-face providers of the DPP, these original pre-existing programmes were usually consumer-facing digital programmes and not specifically related to the face-to-face version of the DPP. Uniquely for provider 3, its programme was described as developed specifically for the pilot DDPP, based on experience of other digital health interventions; such experience highlighted the need for intensive support in the early stages of the programme.

Interviews also identified that the digital programmes are continually improved and developed based on user experience feedback and research conducted by providers. Interviews with developers from provider 3 indicated that the option for group support was introduced after the provider had run its own research to explore user experience of group support.

### Strengths and limitations

All documentation supplied by providers (including transcripts of interviews with health coaches) has been reviewed systematically using a standardised TIDieR checklist. Our decision to adapt the TIDieR checklist for describing the delivery of the digital programme is in line with previous research [27] that suggested TIDieR could not capture the full complexity of an online structured education programme for T2DM. The adaptations to the TIDieR checklist to facilitate detailed description of features of digital delivery is an important innovation that could be used again in future studies, and in itself could inform future iterations of TIDieR for use in describing digital health interventions. By using a mixed methods study design, qualitative data from programme developers has been used to provide important background and context to explain the findings from the document review.

However, it should be noted that participants who were interviewed for this study were not always necessarily directly involved in the development of the provider programmes. It is possible that some people involved in the early stages of design and development had since moved on to other roles. Nonetheless, the research team tried to identify at least one relevant individual from each digital provider and aimed to interview professionals from different backgrounds to gather a range of views and provide a comprehensive understanding of the processes involved in the design and development of each NHS-DDPP intervention. Every effort was made by the research team to obtain access to all relevant documentation for the study but it is possible that we were not given access to all relevant design and delivery documentation by providers (for example we were not provided with the app for provider 3). Content analysis of interviews with health coaches (as key deliverers of the programme) was included in this study to help to fill any potential gaps in information on provider programmes. By conducting triangulation across multiple sources of information in this way, we can be more confident of the overall picture in results.

### Relationship with other literature

Our finding that provider programmes were designed with relatively high fidelity to the NHS service specification is comparable to outputs from an evaluation of the face-to-face version of the NHS-DPP. The NHS-DPP programme design demonstrated good fidelity to the structural features itemised in the programme specification [22]. A similar evaluation of the pilot NHS Low Calorie Diet Programme (which aims to achieve T2DM remission), which is also delivered by multiple providers working to a single service specification, demonstrated relatively good fidelity to the service parameters stipulated in the NHSE specification [28].

The resulting variation in service delivery across providers that is highlighted in this study is significant, particularly because a substantial component of the variation is around features of the programme that provide support to participants. Programmes did have fidelity to the service specification because the NHS specification documentation simply refers to ‘consider social and psychological support needed to support people to implement behaviour changes and to provide individual 1–1 support’ [12] and is not prescriptive about the format of delivery of support or its dose or scheduling over 9 months. This is notable as prior research [29] suggests that the variation in intensity of support offered to participants could be important. In this systematic review of internet-based interventions to promote health behaviour change, effectiveness was enhanced by the use of additional methods of communicating with participants such as: access to an advisor to request advice, scheduled contact with advisor, and peer-to-peer access (eg, peer-to-peer forums or live chat). Accordingly, one might speculate that the exact intensity, quality and nature of support offered to NHS-DDPP participants could be an important factor in its effectiveness. This is also in line with recent findings from a qualitative study of NHS-DDPP participants [19] that concluded that support from health coaches is very much valued and is furthermore instrumental in helping participants understand and use key behaviour change content in the programme. It has also been suggested that professional support features (such as remote contact with a clinician) can positively influence engagement with digital behavioural interventions [30].

To the authors’ knowledge, the concept of repurposing or adapting a pre-existing intervention to meet a service specification, has not been previously evaluated in the literature. Accordingly, there is some degree of uncertainty regarding the implications of this. Knowledge of this route of programme development could, to some extent, explain the lack of theoretical underpinning for the programme demonstrated previously [17]. In a situation where pre-existing programmes are adapted to meet a service specification, even if there is clear theoretical basis for the pre-existing programme, there is a risk of lack of clarity about how the programme is expected to work when the end purpose of the programme changes to meet a specification (for example from personal health coaching to T2DM prevention). As previously discussed [17], this lack of clarity risks effective translation of behaviour change content in intervention design to intervention delivery.

Our finding that shows the NHS-DDPP is continually updated and reviewed by providers, based on user feedback, is positive. An expert consensus [31] has highlighted the need for a user-centred and iterative approach to development of digital interventions, to progressively refine the intervention to meet user requirements. Nevertheless, there is potential for drift in service delivery of this national programme, based only on the needs of active users of the programme rather than taking account of the needs of people who have difficulty taking up and engaging with the NHS-DDPP.

The current study is part of a research programme that provides a thorough fidelity investigation of the NHS-DDPP, and we are not aware of similar work on any other nationally-implemented diabetes prevention programmes (DPPs). It is therefore impossible to directly compare our findings to fidelity assessments of DPPs in other high-income countries. However, fidelity of nationally implemented DPPs is likely to be an issue because large-scale programmes sometimes commission several different providers (private, state or third sector) to deliver the programme on their behalf, following central guidance, with some room for interpretation [18]. These issues may be particularly pertinent for longer-term programmes. It is therefore plausible that the issues identified in the current study, particularly around important variation in structural features of the programme delivered across providers, may be applicable in other countries.

### Implications for research

An immediate question for future research is understanding whether the variation in delivery of the NHS-DDPP across providers is related to any differences in health outcomes (HbA1C and/or weight loss). It would be helpful to know whether participants who receive more support through the programme are more likely to achieve behaviour change and prevention of T2DM, and whether there are particular population groups that benefit most from this support. To date, we are not aware of any analyses of the effectiveness of the implemented NHS-DDPP that report health outcomes according to providers, but would urge investigators and research funders to consider this as a next step. Experiences from an analysis of the face-to-face DPP lend support to this premise, as large differences in health outcomes were reported across providers, and such variation was found to be a more important factor on outcomes than variation in patient characteristics [8].

### Implications for practice

This work has potential implications for the NHS-DDPP and the way in which the service is commissioned. NHSE appear to have used a pragmatic approach to implement the NHS-DDPP at pace and at scale, using multiple independent providers to deliver the service. Providers’ approach of adapting pre-existing programmes to meet the NHS-DDPP specification has advantages in terms of meeting a service need efficiently. Recent findings suggest that the face-to-face version of the programme achieves a reduction in population incidence of T2DM [32] and that a digital version of the service can be just as effective [11]. It could be argued that a benefit of this commissioning model is the opportunity to harness expertise already developed by commercial providers, for example around experiences in delivering health coaching. Continuing with this model for commissioning the service allows innovation from commercial providers to be capitalised upon. Notwithstanding this success, there is room for improvement in commissioning of future rounds of the service. It is recommended that future rounds of commissioning include clearer specifications for the type of support participants should receive and detail about the expected dose and scheduling of this support. We acknowledge the potential for tension between supporting fidelity of the national programme with a rigid service specification versus the need for flexibility to allow continual improvement of the programmes through user feedback, and suggest programmes are continually monitored to identify any significant drift in service delivery. It is important that such monitoring also considers the relationship with health outcomes to inform understanding of the most effective components within the programme. The research team continue to share findings from this programme of research with NHSE and a number of improvements have already been made [18].

## Conclusion

Provider plans for the NHS-DDPP show relatively high fidelity to the NHS service specification. Despite this, there was wide variation in structural features of delivery of the NHS-DDPP across providers. This was most evident for delivery of ‘support’, in terms of use of health coaching and/or group support, with further variation across delivery channel and method, and dose and scheduling. Interviews with developers of the provider programmes showed that much of this variation is likely to be attributable to the ways in which the provider programmes were developed. The origin of each provider’s programme was usually a pre-existing programme that was adapted to become in line with the NHS service specification for the DDPP, and much of the ethos of the original programmes remained intact. Each provider version of the NHS-DDPP is continually improved and developed based on user experience feedback and research conducted by the providers.

## Electronic supplementary material

Below is the link to the electronic supplementary material.

Supplementary Material 1

Supplementary Material 2

Supplementary Material 3

Supplementary Material 4

## Data Availability

The materials from digital providers and audio-recordings of interviews analysed in the current study are not publicly available due to confidentiality agreements with the provider organisations, as some information is commercially sensitive. Some datasets are available from the corresponding author on reasonable request, although authors will require the explicit permission of the relevant provider organisations.

## List of abbreviations

BCT: Behaviour Change Technique
DDPP: Digital Diabetes Prevention Programme
DPP: Diabetes Prevention Programme
HbA1c: Glycated haemoglobin (used as a measure of blood sugar levels over several months)
HC: Health Coach
NHS: National Health Service
NHSE: National Health Service England
NICE: National Institute of Health and Care Excellence
TIDieR: Template for Intervention Description and Replication
T2DM: Type 2 Diabetes Mellitus
