# Supplementary material for: Description of the nationally implemented National Health Service digital diabetes prevention programme and rationale for its development: mixed methods study
Source: BMC Health Serv Res. 2023 Apr 18;23:373. doi: 10.1186/s12913-023-09210-3 (PMC10114366; doi:10.1186/s12913-023-09210-3)
Supplement: Supplementary file 1 — Supplementary Material 1 [file 12913_2023_9210_MOESM1_ESM.docx]

**Additional file** **1: Programme delivery materials obtained from each of the four digital providers**

|  | **Provider 1** | **Provider 2** | **Provider 3** | **Provider 4** |
| --- | --- | --- | --- | --- |
| **Delivery materials analysed** | | | | |
| **Content included** |  |  |  |  |
| Access to app / online educational content | **✓** | **✓** | **✓** | **✓** |
| Educational leaflets / workbooks | **✓** | **✓** | n/a | **✓** |
| **How content was assessed** |  |  |  |  |
| Access to app / online educational content | App access; Online articles unlocked daily over 12 weeks, plus 8 optional 4-week courses | App access | 42 online modules; App user guide obtained | App access; Weekly online articles |
| Educational leaflets / workbooks | Nutritional handbook and recipe book | PDF documents, videos and links sent to service users | All educational content was accessed via online modules. Printed alternative not supplied | NDPP handbook |
| **Transcripts analysed** | | | | |
| No. of interviews with health coaches employed by each provider | 2 | 4 | 2 | 4 |
