# Supplementary material for: Description of the nationally implemented National Health Service digital diabetes prevention programme and rationale for its development: mixed methods study
Source: BMC Health Serv Res. 2023 Apr 18;23:373. doi: 10.1186/s12913-023-09210-3 (PMC10114366; doi:10.1186/s12913-023-09210-3)
Supplement: Supplementary file 2 — Supplementary Material 2 [file 12913_2023_9210_MOESM2_ESM.docx]

**Additional file** **2: Interview schedule for interviews with health coaches**

**Evaluating the Digital NHS Diabetes Prevention Programme**

**Question schedule**

Thank you for agreeing to take part in this interview. Please let me know if you would like to take a break or pause the recording at any point, or if you would like to stop the interview. Please note the information we collect will be kept securely and confidentially in accordance with data protection law, as described on the participant information sheet. The interview should take no more than one hour.

**Questions about your day-to-day role as a Health Coach**

1. How long have you worked for [digital provider]?
2. How did you get into this/ what was your previous role/ background?
   1. What skills or expertise did you bring?
   2. What is your previous experience of delivering behaviour change interventions or personal coaching (if any)?
   3. Were you employed by [digital provider] before they won a contract to deliver the NHS DDPP? If yes, how does your role differ now from when you were delivering the pre-existing [digital provider] programme?
3. Could you walk me through what your ‘typical day’ as a Health Coach looks like?
   1. What is your first job of the day?
   2. What general roles and responsibilities do you have as a Health Coach?
   3. Does your day involve much interaction with other Health Coaches?
4. Are you involved in delivering initial assessment calls at the beginning of the programme/ discharge calls at the end of the programme?
   1. Could you walk me through what a typical phone call would look like?
   2. E.g. what topics do you cover? What do you deliver to support behaviour change during this phone call?
5. Do you have any other additional responsibilities as a Health Coach?
   1. E.g. training, mentoring, quality assurance?
6. Could you tell me a bit about the general support that you offer to service users?
   1. Telephone support?
   2. Support via one-to-one messages?
   3. Signposting?

**(Elicit description of components, then in turn):**

- **Can you give me an example of this type of support that you would typically offer?**
- **How far through the 9 month programme do you offer this support? How frequently?**
- **How does your support change (or not) as participants go through the programme?**
- **Do you think service users benefit from this type of support?**
  - **How do they benefit from this support? / Why do you think this type of support is not as effective?**

1. Does your role involve moderating the virtual support networks that service users can join?
   1. Can you give me an example of this type of support that you would typically offer?
   2. Do you think service users benefit from this type of peer-to-peer support?
      1. How do they benefit from this support? / Why do you think this type of support is not as effective?
2. How do you tailor or personalise your coaching to the individuals taking part?
   1. What personal characteristics do you consider?
   2. Can you give some examples of how you might adapt your coaching techniques?
3. Do you provide any educational material/content to service users on the programme to increase their knowledge or understanding of pre-diabetes?
   1. What educational content do you deliver? (E.g. diet and PA recommendations, information about pre-diabetes)
   2. What format is this content typically delivered? (E.g. over the phone, sending videos, emailing articles, responding to questions?)
4. Could you tell me a bit about the behaviour change support you offer to service users on [digital provider’s] programme to manage their own health?
   1. Goal setting?
   2. Action planning?
   3. Self-monitoring?
   4. Support and encouragement?
   5. Feedback?
   6. Problem solving?

**(Elicit description of components, then in turn):**

- **Can you give me an example of this type of behaviour change support you would typically offer? (E.g. what behaviours would this target?)**
- **In what format do you deliver this support? (E.g. telephone, email, messenger)**
- **How often do you offer this type of behaviour change support to an individual?**
- **Do you think this type of support works in helping service users to change their behaviour?**
  - **How do they benefit from this behaviour change support? / Why do you think this type of support is not as effective?**

1. How confident do you feel in delivering this type of behaviour change support?
   1. Goal setting?
   2. Action planning?
   3. Self-monitoring?
   4. Support and encouragement?
   5. Feedback?
   6. Problem solving?
2. What training have you received from [digital provider]?
   1. When was this training?
   2. How often are you expected to complete training?
   3. Did this training meet your needs?
   4. Are there any aspects of your job role where you feel you would benefit from additional training from [digital provider]?
3. Have there been any recent modifications to the NHS-DPP digital programme that have impacted on your role as Health Coach? If so, how?
4. What are your general experiences of delivering this diabetes prevention programme digitally?
   1. What are your positive experiences?
   2. How could the programme be improved?
5. Is there anything else about your role as a Health Coach that we haven’t covered today and you want to talk about?

*Thank you very much for taking the time to take part in our research. This is our final interview with you.*
