# Supplementary material for: Description of the nationally implemented National Health Service digital diabetes prevention programme and rationale for its development: mixed methods study
Source: BMC Health Serv Res. 2023 Apr 18;23:373. doi: 10.1186/s12913-023-09210-3 (PMC10114366; doi:10.1186/s12913-023-09210-3)
Supplement: Supplementary file 3 — Supplementary Material 3 [file 12913_2023_9210_MOESM3_ESM.docx]

**Source document (title, version, date):**

**Researcher:**

**Date completed:**

**Aim**

To describe all the elements of the digital NHS DPP intervention, as specified by each of the four digital providers which will be delivered via their online platform.

**Instructions for TIDieR coding**

Keep notes of any queries or problems with the extraction process.

**(1) Name:** ‘NHS Digital Diabetes Prevention Programme’ and specify the name of the provider.

**(3) Materials (‘what’):**

- List the materials used during the individual assessment before participants are recruited onto the programme under a sub-heading ‘*Initial Assessment*’.
- If stated, list the resources used in the intervention under heading ‘*Virtual Materials*’
  - Includes educational videos, external website, e-learning modules. Include summary of topics.
  - For apps, specify key functions such as setting goals, tracking weight & behaviours
- If there is the option for any physical materials to be sent to users’ homes, specify these under a separate sub-heading: ‘*Physical Materials*’ (e.g. recipe books sent to users’ homes, DVDs, weighing scales, wearables etc.)

**(4) Procedures (‘what’):**

- List the procedures in the order they appear in the source document.
- Procedures include separate activities that the service users will carry out during the intervention.
- Top-line detail of main functions of intervention presented here, cross reference to items 7 & 9 where detail will be e.g. if HC calls are completed at particular milestones.

**(5) Deliverers (‘who’):** Describe the health coach/facilitator delivering any component of the intervention:

- Their job title (eg Health Coach, dietitian);
- Their expertise/qualifications (specify if healthcare professional, nutritionist, dietitian);
- Any specific training they undertook to deliver this intervention;
- Whether their competence in delivering the DPP was assessed/monitored throughout the intervention.

**(6) Format of delivery (‘how’):**

For each ‘procedure’ listed under 5, break down into:

***Mode of delivery*** (face-face, remote, face-face and remote, environmental prompts

Include video calls from HC as ‘face-face and remote’. Use environmental prompts for materials sent to service user.

***Delivery method*** (individual or group, including group size where relevant)

***Delivery channel*** (App, chat messaging, text message, email, telephone call, podcast, video, website)

Include e-learning modules as website. For each channel specify whether *Interactive* or *Passive,* to describe whether it is a one-way or two-way exchange eg an automated email is passive, online chat with HC interactive

***Delivery route*** (Audio, text, picture, experiential, unclear)

Eg specify if website/e-learning is text, picture or both. Experiential would cover app usage

**(8) Dose & Scheduling (‘when and how much’):**

Breakdown into *Health Coaching* and *Sessions (e-learning, website)*

- ***Sessions***
- Specify the number of ‘core’ and ‘maintenance’ sessions and specify the schedule of when they are delivered over the course of the intervention (e.g. weekly, monthly).
- Specify the duration of each online session if applicable (e.g. “Week 1: Core session one [30 minutes]”) and the duration of the overall intervention.
- ***Health coaching***
- Specify number, duration and frequency of contacts/ communications with Health Coaches
- Specify any change to dose/availability of health coaching over 9 months (eg HC stops at 12 weeks, or reduction in frequency of calls at 6 months)
- Specify contact form – scheduled, proactive (initiated by HC), reactive (initiated by SU), random or unclear

**(9) Tailoring:**

- Describe any adaptations that were made to the digital DPP sessions (e.g. the use of any culturally relevant materials, tailoring information provision in accordance with health literacy of service users).
- Describe why and when these adaptations were made.
- Include description of any materials that are personalised to individuals, specifying whether *automated* eg email feedback, and/or *tailored by whom (SU or HC)*

**(10) Modifications:**

- If the intervention was modified during the course of the study, describe the changes (what, why, when and how)

| **TIDieR item (TIDieR item no.)** | **Location** | **Extracted information** | **Comments, highlighting any potential modifications (10)** |
| --- | --- | --- | --- |
| **Name (1)** or brief description of intervention |  |  |  |
| **Materials (3)**  Physical, informational or digital materials; provided to participants or used in intervention delivery  *Initial assessment*  *Virtual materials*  *Physical materials* |  |  |  |
| **Procedures (4)**  Procedures, activities, and/or processes used in intervention, including enabling or support activities |  |  |  |
| **Deliverers (5)**  Expertise, background, any specific training |  |  |  |
| **Format of delivery (6)**  *Mode of delivery* (remote, face-face etc..)  *Delivery method* (group, individual etc)  Delivery channel (app, chat, email, website etc…..include whether *Interactive* or *Passive)*  *Delivery route* (audio, text etc..) |  |  |  |
| **Dose & scheduling (8)**  Number of times the intervention delivered, over what period of time, number of sessions, schedule, duration, intensity and/or dose  *Sessions (details of core, maintenance)*  *Health coaching (scheduling, frequency, duration over 9 mo, including whether proactive/reactive)* |  |  |  |
| **Tailoring (9)**  If intervention to be personalised, titrated or adapted, describe what, why, when, and how  *Automated?*  *Tailored by whom?* |  |  |  |
|  |  |  |  |
