# Supplementary material for: Description of the nationally implemented National Health Service digital diabetes prevention programme and rationale for its development: mixed methods study
Source: BMC Health Serv Res. 2023 Apr 18;23:373. doi: 10.1186/s12913-023-09210-3 (PMC10114366; doi:10.1186/s12913-023-09210-3)
Supplement: Supplementary file 4 — Supplementary Material 4 [file 12913_2023_9210_MOESM4_ESM.docx]

**Additional file 4: Interview schedule for interviews with programme developers**

**Evaluating the Digital NHS Diabetes Prevention Programme**

**Question schedule**

Thank you for agreeing to take part in this interview. Please let me know if you would like to take a break or pause the recording at any point, or if you would like to stop the interview. Please note the information we collect will be kept securely and confidentially in accordance with data protection law, as described on the participant information sheet. The interview should take no more than one hour.

**Questions about designing the digital offering of the ‘Healthier You’: Diabetes Prevention Programme**

1. What is your professional background?
   1. What is your previous experience of developing digital interventions (if any)?
2. How long have you worked for [Provider/ Digital partner]?
3. How did [Provider] and [Digital partner] work together to adapt the current NHS DPP to be a digital offering?
   1. How did [Provider] and [Digital partner] develop a collaboration?
   2. What was [Provider’s] role in the development of the digital offering?
   3. What was [Digital partner’s] role in developing the digital offering?
4. What was your role in the development of the digital NHS DPP intervention?
   1. Involved in commissioning process?/ Involved in design of IT system?/ Involved in refinement of specific aspects of the intervention?
   2. Who else did you work with?
   3. When did your involvement stop/ start?
5. Could you explain a little about how the intervention is made available to people at risk of developing Type 2 diabetes?
   1. How is the intervention framed/presented to people at risk of type 2 diabetes?
   2. Why do you think people would choose to engage with this particular intervention?
6. What strategies are in place to engage and/or support people at first contact with the intervention (onboarding strategies)?
   1. Is there anything in place to address the potential digital divide in terms of access to, use of, or impact of the intervention/ support those less familiar with digital interventions?
   2. What strategies or intervention features are there to encourage on-going use/avoid dropout?
7. Was the digital DPP intervention adapted from a pre-existing digital intervention?
   1. What was the purpose/aim of the original digital intervention?
   2. What changed?
   3. What were the reasons for this change?
   4. In retrospect, was this a good idea? What was lost/ gained by making these changes?
8. How was the digital DPP intervention changed/ adapted from the face-face DPP intervention?
   1. What changed?
   2. What were the reasons for this change?
   3. In retrospect, was this a good idea? What was lost/ gained by making these changes?
   4. How would you describe the relationship between the face-face DPP intervention and the digital DPP intervention? Is any form of integration designed to take place between the two?
9. What theoretical principles about how to change behaviour were involved in developing the digital intervention?
   1. What were these?
   2. How familiar with these ideas were/ are you?
   3. Were there any explicit models of why the intervention should work?
      1. Was this explained in any documents you used/ had access to?
   4. Was a logic model developed?
   5. Was there anyone you worked with who provided this kind of theoretical input?
10. What is your understanding of why the intervention should be helpful for people who are at risk of developing Type 2 diabetes?
    1. In what ways should it help them?
    2. How does the intervention actually help with this?

**(Elicit components and description, then in turn):**

- - **What was this component trying to achieve?**
  - **Do you think it achieved that? Why?**
  1. Do you have any expectations in relation to how much use/engagement of the intervention is required to have the desired effect?

1. What content did you include in the digital intervention?
   *Try to elicit answers for the following:*
   1. Increasing knowledge/understanding of pre-diabetes
   2. Changing behaviour, e.g. what were the behaviours you were targeting?
   3. Managing own health
   4. Managing feelings and emotions

**(Elicit description of components, then in turn):**

- 1. **In what ways should this component help people?**
  2. **Do you think it achieved that? Why?**
  3. **Did you change or adapt this content for the digital intervention?**
     1. **What were the reasons for this?**
     2. **What was lost/ gained by making these changes?**

1. What behaviour change techniques (/techniques/tools to help with lifestyle change) did you include in the digital intervention? [*Will need to explain what this entails*]:
   1. Providing information about health consequences
   2. Goal setting
   3. Action planning
   4. Reviewing goals
   5. Problem solving
   6. Prompting self-monitoring
   7. Providing feedback on performance

**(Elicit description of components, then in turn):**

- 1. **Which behaviours was this technique/BCT targeting?**
  2. **What was this technique/BCT trying to achieve? Why do you think it will work?**
  3. **Do you think it achieved that? Why?**
  4. **Did you have to change or adapt this technique/BCT for the digital intervention?**
     1. **What were the reasons for this?**
     2. **What was lost/ gained by making these changes?**

1. What content did you include in the digital intervention to support engagement?
   *Possible answers could include the following:*
   1. Interactive activities?
   2. Reading material?
   3. External websites?
   4. Worksheets?
   5. Videos?

**(Elicit description of components, then in turn):**

- 1. **In what ways should this content help people?**
  2. **Do you think it achieved that? Why?**
  3. **Was this content adapted or changed from the face-to-face (or pre-existing digital intervention) to the digital intervention?**
     1. **What were the reasons for this?**
     2. **What was lost/ gained by making these changes?**

1. What support networks did you include in the digital intervention?
   *Possible answers could include the following:*
2. One-to-one coaching? *[Most interested in this]*
3. Group support? *[Most interested in this]*
4. External websites?
5. Signposting?
6. Videos?

**(Elicit description of components, then in turn):**

- 1. **In what ways should this component help people?**
  2. **Do you think it achieved that? Why?**
  3. **How does this type of support differ to that delivered in the face-to-face intervention?**

1. Is there anyone else who you think was important in the development or refinement of the intervention?
   1. Was there any Patient and Public Involvement (/service user testing/patient involvement) in the development stages?
2. Is there anything else about the process of developing/ adapting the intervention that was important?

*Thank you very much for taking the time to take part in our research.*
